# The Sickle Cell Disease Functional Assessment (SCD-FA) tool: a feasibility pilot study

**DOI:** 10.1186/s40814-022-01005-3

**Published:** 2022-03-04

**Authors:** Charity I. Oyedeji, Katherine Hall, Alison Luciano, Miriam C. Morey, John J. Strouse

**Affiliations:** 1 Department of Medicine, Division of Hematology, Duke University School of Medicine, Durham, NC USA; 2 Duke Claude D. Pepper Older Americans Independence Center, Durham, NC USA; 3 Department of Medicine, and Duke Comprehensive Sickle Cell Center, Duke University School of Medicine, Durham, NC USA; 4 Department of Medicine, Division of Geriatrics, Duke University, Durham, NC USA; 5 Geriatric Research, Education and Clinical Center, Durham Veterans Affairs Medical Center, Durham, NC USA; 6 Division of Pediatric Hematology-Oncology, Duke University, Durham, NC USA

**Keywords:** Sickle cell disease, Functional assessment, Gait speed, Geriatric assessment, Aging, Frailty, Older adults, Geriatrics

## Abstract

**Background:**

The life expectancy for individuals with sickle cell disease (SCD) has greatly increased over the last 50 years. Adults with SCD experience multiple complications such as cardiopulmonary disease, strokes, and avascular necrosis that lead to limitations that geriatric populations often experience. There are no dedicated instruments to measure functional decline and functional age to determine risk of future adverse outcomes in older adults with SCD. The objective of this study was to assess the feasibility of performing the Sickle Cell Disease Functional Assessment (SCD-FA).

**Methods:**

We enrolled 40 adults with SCD (20 younger adults aged 18–49 years as a comparison group and 20 older adults aged 50 years and older) in a single-center prospective cohort study. Participants were recruited from a comprehensive sickle cell clinic in an academic center in the southeastern United States. We included measures validated in an oncology geriatric assessment enriched with additional physical performance measures: usual gait speed, seated grip strength, Timed Up and Go, six-minute walk test, and 30-second chair stand. We also included an additional cognitive measure, which was the Montreal Cognitive Assessment, and additional patient-reported measures at the intersection of sickle cell disease and geriatrics. The primary outcome was the proportion completing the assessment. Secondary outcomes were the proportion consenting, duration of the assessment, acceptability, and adverse events.

**Results:**

Eighty percent (44/55) of individuals approached consented, 91% (40/44) completed the SCD-FA in its entirety, and the median duration was 89 min (IQR 80–98). There were no identified adverse events. On the acceptability survey, 95% (38/40) reported the length as appropriate, 2.5% (1/40) reported a question as upsetting, and 5% (2/40) reported portions as difficult. Exploratory analyses of physical function showed 63% (25/40) had a slow usual gait speed (< 1.2 m/s).

**Conclusion:**

The SCD-FA is feasible, acceptable, and safe and physical performance tests identified functional impairments in adults with SCD. These findings will inform the next phase of the study where we will assess the validity of the SCD-FA to predict patient-important outcomes in a larger sample of adults with SCD.

## Key messages regarding feasibility

What uncertainties existed regarding the feasibility?

- The importance of early identification of functional impairment has been well-established in geriatric populations without sickle cell disease (SCD). Currently, the six-minute walk test is the only objective measure routinely used to assess function in individuals with SCD. Little is known about the feasibility and acceptability of using geriatric assessment measures to evaluate function in adults with SCD.

2) What are the key feasibility findings?

- The key feasibility findings of this work are that the Sickle Cell Disease Functional Assessment (SCD-FA) is feasible, acceptable, and safe with 80% of approached patients consenting to participate, 91% completing the SCD-FA, and nearly all participants reporting no difficulties performing and understanding the SCD-FA measures.

3) What are the implications of the feasibility findings for the design of the main study?

- The findings of this study provide evidence for optimizing the SCD-FA into a briefer assessment and will inform the design of future research. We plan to assess the validity of the SCD-FA to predict patient-important outcomes in a larger sample of adults with SCD and develop interventions to address functional deficits identified by the SCD-FA.

## Background

Survival for individuals with sickle cell disease (SCD) has substantially improved over the last 50 years with median survival increasing from 14 years, based on autopsy data reported by Diggs in 1973, to approximately 40 to 45 years in population-based studies and 61 years in cohorts recruited at comprehensive programs [1–3]. Thirteen percent of the adults cared for in our sickle cell center are 50 years or older. As individuals with SCD age, they demonstrate substantial and early deterioration of multiple organ systems due to hemolysis, vaso-occlusion, and downstream sequelae [4]. This often leads to complications seen in other geriatric populations, such as cardiopulmonary disease, functional decline, and cognitive impairment [4–6]. Despite improvements in survival, there are minimal data on the appropriate care for older adults with SCD, and there are no standardized, validated instruments to measure function and detect vulnerabilities in this aging population.

### Aging and SCD

Many experts define aging as a deterioration in physiological function that occurs over time [7, 8]. The World Health Organization defines healthy aging as the process of maintaining functional ability in older age, which includes the ability of individuals to be and do what they value [9]. Chronologic age does not accurately reflect health and functional status [10]. Individuals with similar chronologic age often have different functional capabilities and estimated life expectancy [11]. SCD and age-related changes can lead to impairments in function and disability. If functional impairments are identified early, healthcare providers can implement interventions to reduce functional decline and improve quality and quantity of life [12]. Failure to identify impairments early can lead to adverse outcomes, such as death, loss of independence, and a diminished ability to do activities and meet goals they value most in life. Therefore, establishing a patient’s baseline function will facilitate monitoring the trajectory of their health [13].

### Current functional assessment tools in SCD

Current validated assessment tools used to evaluate health and function in adults with SCD are limited in what they are able to measure. The only physical performance test routinely used to assess patients with SCD is the six-minute walk test (6MWT), a measure of cardiorespiratory function and screening tool for pulmonary hypertension [14]. It has limited utility as it does not include assessments of strength or balance, which are factors that are especially vital to maintaining health, independence, and function in late life. Available condition-specific assessment tools, such as the Adult Sickle Cell Quality of Life Measurement System, ASCQ-Me, provide patient-reported data about important areas of health in adults with SCD, such as mental health, pain, sleep, and fatigue. However, these measures are limited in their ability to identify age-related conditions, such as frailty and dependence in activities of daily living and instrumental activities of daily living (ADL/IADL) [15].

### Comprehensive Geriatric Assessment

Comprehensive Geriatric Assessment (CGA) is a process used to assess the capabilities and vulnerabilities in older adults and can be used to assess an individual’s physiologic or functional age. It measures multiple domains of health including functional status, physical performance, cognitive function, psychological state, nutritional status, social support, comorbidities, and polypharmacy [16]. The CGA gives a more accurate representation of a patient’s ability to adapt to the demands of daily life and can unearth unsuspected impairments in function often missed by routine clinical evaluations [10]. Geriatricians have used CGA for decades to guide therapeutic interventions. Oncologists most notably use CGA to assess risk of toxicity from chemotherapy in older adults and have also included younger adults with hematologic malignancies in these studies [10, 17, 18].

### Study objectives

The goal of this research was to apply geriatric functional assessment measures to older adults with SCD. In this pilot study, we assessed the feasibility of performing the Sickle Cell Disease Functional Assessment (SCD-FA) in adults with SCD. Our rationale for developing the SCD-FA was to create a standard method for assessing risk of adverse outcomes, address the unique needs of the growing population of older adults living with SCD, and identify modifiable deficits to guide development of interventions to improve function and quality of life and reduce frailty and mortality.

## Methods

### Patients and study design

We enrolled 20 older adults (age ≥ 50 years) in a prospective cohort study to assess the feasibility of a functional assessment for SCD. We later added an additional 20 younger adults (age 18–49 years) as a comparison group to determine if younger adults performed better on the SCD-FA compared to older adults given the progressive nature of SCD. Participants were enrolled from October 2018 to July 2020. Enrollment continued until we reached the target of 40 participants. We recruited participants from a single academic medical center in the Southeastern United States. We included participants that (1) have a diagnosis of SCD confirmed by hemoglobin electrophoresis, high-performance liquid chromatography (HPLC), or genotyping and (2) speak fluent English. We excluded patients if they (1) were previously diagnosed with moderate to severe cognitive impairment by their usual outpatient providers, (2) were unable to self-consent, or (3) were wheelchair-bound at the time of recruitment. Participants were screened for eligibility by chart review and approached during routine clinic or sickle cell day hospital visits. No potential participants that were approached had to be excluded for cognitive impairment, inability to consent, language barriers, being wheelchair-bound, or inability to reach steady-state. The study was approved by the institutional review board. All participants provided written informed consent prior to enrollment.

### Data collection

Participants entered their demographics, complications from SCD, social history (e.g., education, annual household income, living arrangements, employment status), and healthcare utilization into a REDCap database. Investigators also recorded SCD complications, healthcare utilization, and steady-state laboratory values from the electronic medical record. All participants were assessed at steady-state, which we defined as greater than 6 weeks after their last hospitalization and greater than 2 weeks after their last emergency department (ED) or sickle cell day hospital visit. We confirmed participants were at steady-state by reviewing the medical record and asking the participant. We managed the data collection using REDCap electronic data capture tools [19, 20]. We included consensus measures from the Phenotypes and eXposures (PhenX) Toolkit when available [21]. Conflicts between self-report and investigator entered observations were handled by using medical history and healthcare utilization as documented in the electronic medical record.

### Description of measures in the SCD-FA

The SCD-FA includes a combination of validated patient-reported questionnaires and performance-based measures administered by the study team (Table 1). This assessment focuses on key areas that are at the intersection of SCD and geriatrics collaboratively selected by SCD providers, geriatricians, and exercise specialists. We included measures from the oncology geriatric assessment developed by Hurria et al. [16, 17]. Measures were originally selected based on validity, brevity, reliability, and prognostic value [16, 17].

**Table 1:** Measures selected for the sickle cell disease functional assessment

Domains	Measures in each domain	Description
Functional Status	OARS Activities of Daily Living and Instrumental Activities of Daily Living	Measures the extent to which one is able to function independently. Scores range 0–28 with higher scores indicating more independence. (14 items)
	Physical Functioning Subscale of the SF-36	Component of the 36-Item Short Form Health Survey (SF-36). Measures extent to which health currently limits daily activities. Scores range 0–100, with higher scores indicating better physical functioning. (10 items)
	Karnofsky performance status—self and physician*	Global measure of physical ability. Scores 0–100 with higher scores indicating better performance (2 items). Score ≥ 80 indicates good performance status.
	Number of falls in last 6 months	Self-reported number of falls in the last 6 months. ≥ 1 fall indicates increased risk of subsequent falls. (1 item)
	Usual Gait Speed*	Comfortable walking pace on a 3-m (10 ft) course with an acceleration zone and deceleration zone. The fastest speed of 2 trials is used for the analysis. Scores compared to normative values based on age and gender.
	Timed Up and Go*	The time it takes to rise from a standard height chair (46cm), walk a distance of 10 ft (3 m), turn, walk back to the chair, and sit down again. Shortest time of 2 trials is used for the analysis. TUG assesses balance. TUG > 10 s is associated with fractures in older adults and > 12 s indicates increased risk of falls.
	Dual-Task Performance*	Assesses the effects of simultaneously performing a cognitive and motor task: usual walking speed for 1 minute and a verbal fluency task using letters F, A, and S. Each task is performed once individually, then simultaneously twice using a different letter on each attempt. Scores are used to calculate whether cognitive-motor interference is present.
	Six-Minute Walk Test*	Distance walked in 6 minutes. 2-min and 6-min walking distances are recorded and heart rate recovery at 1 and 2 minutes. Scores compared to normative values based on age and gender.
	Seated Grip Strength*	Grip strength measured in triplicate alternating both hands using Jamar Technologies Hydraulic Hand Dynamometer while the participant remains seated in a standard height chair (46 cm). Scores compared to normative values based on age and gender.
	30-second Chair Stand*	Number of times one can rise to a standing position and sit back down in 30 seconds. Scores compared to normative values based on age and gender.
Comorbid Medical Conditions	OARS Physical Health questionnaire	Patient-reported comorbidity checklist and the degree to which the condition interferes with their daily activities. (27 items)
Psychological State	Mental Health Inventory-18 (MHI-18)	Includes anxiety, depression, behavioral control, and positive affect subscales. Total and subscale scores range 0–100, with higher scores indicating better mental health. (18 items)
Social Support/Social Activities	MOS Social Support Survey	Perceived availability of social support. Total score is on a 0–100 scale with lower scores indicating less support. (19 items)
	Social Functioning Subscale of the SF-36	Measures amount of time physical health or emotional health interfered with social activities over the last 4 weeks. Subscale scores range from 0-100 with higher scores indicating better social functioning. (1 item)
	MOS Social Activity Limitations Measure	Measures change in social activities and limitations in social activities compared to peers over the last 6 months. Mean of 2 items transformed to scores range 0–100 with higher scores indicating less limitations. (2 items)
Nutritional Status	Body Mass Index	Calculated by measuring height and weight. A low BMI for older adults is < 22 kg/m^2^ and obesity is ≥ 30 kg/m^2^. (1 item)
	Unintentional Weight Loss	Unintentional weight loss in the last 6 months. Unintentional weight loss > 5% in 6–12 months is associated with increased mortality. (1 item)
Cognition	Blessed Orientation-Memory-Concentration Test*	Measures temporal orientation, short-term memory, and concentration. Weighted score ranges 0-28 with higher scores indicating more impairment. A score > 9 is concerning for cognitive impairment. (6 items)
	Montreal Cognitive Assessment*	Measures visuospatial skills, executive functions, memory, attention, calculation, concentration, language, abstraction, and orientation. On a scale of 0-30 with a score < 26 indicating cognitive impairment. (16 items)
Medications	Comprehensive list of medications	Participant recorded medication list. Polypharmacy is defined as ≥ 5 prescribed medications.

*OARS* Older Americans Resources and Services, *MOS* medical outcome study, *SF*-*36* 36-item Short Form, *TUG* timed up and go, *BMI* body mass index

*Indicates performance-based measures that must be administered by personnel

The 7 domains of the SCD-FA are functional status, comorbid medical conditions, psychological state, social support/social functioning, weight status, cognition, and patient-reported medication list. The oncology geriatric assessment included 3 provider-administered measures: Timed Up and Go (TUG), provider-reported Karnofsky Performance Status (KPS), and the Blessed Orientation Memory Concentration Test. We added 5 provider-administered physical performance measures to the TUG: usual gait speed, 6MWT with heart rate recovery, seated grip strength, 30-second chair stand, and dual-task performance (also includes cognitive component). We compared the results of each physical performance test to normative values based on age and gender where such data were available [22]. We added the Montreal Cognitive Assessment (MoCA) as an additional cognitive measure and the reading subtest of the Wide Range Achievement Test 5^th^ Edition to account for differences in academic achievement beyond stated education level. For a detailed description of each measure, see Table 1 and published protocol [23].

### Administering SCD-FA

Investigators who were SCD physicians received training and ongoing guidance on how to properly perform and analyze the physical function portions of the SCD-FA throughout the study. The SCD-FA was administered by first author and a trained research specialist in a quiet hallway in the sickle cell clinic.

### Outcomes

The endpoints of this study are based on Consolidated Standards of Reporting Trials (CONSORT) extension to pilot and feasibility trials guidelines [24, 25].

### Primary endpoint

The primary endpoint was the proportion of participants who completed the assessment in its entirety out of those that were consented. We defined feasibility as ≥ 80% of participants completing the SCD-FA.

### Secondary endpoints

The secondary endpoints were proportion consenting, duration of the SCD-FA, adverse events, and acceptability. We defined feasibility for these secondary endpoints as (1) ≥ 80% providing written consent of those approached to participate, (2) a mean duration for completing the SCD-FA ≤ 120 min, and (3) no moderate or severe adverse events within 48 h after completing the SCD-FA.

We assessed acceptability of the SCD-FA with a satisfaction survey at the end of each study visit. We included questions about acceptability of the time it took to complete the entire assessment, whether questions were difficult to understand, uncomfortable, or upsetting, and if there were measures that should be added or removed. Acceptability was defined as < 20% reporting difficulties in understanding measures or reporting questions as upsetting or uncomfortable and at least 80% reporting the length of the SCD-FA as appropriate.

### Statistical analysis

We assessed feasibility overall and by age group by calculating proportions for each endpoint and the proportion of participants and individual measures with missing data. We described acceptability survey responses using simple descriptive statistics. Given that the primary purpose of this study is to evaluate feasibility, the remaining analyses were exploratory and descriptive in nature. We used descriptive statistics and visual displays to summarize the demographic data and unadjusted results of the SCD-FA measures. We compared the younger and older participants to age- and sex-matched normative data. We determined an equivalent functional age by the comparing the mean physical performance score for each group by age and gender to expected normative values based on age and gender in the general population [22, 26–29]. Results were not interpreted as definitive in size or direction, or causal in their effect. We conducted analyses in R Statistical Software version 3.6.1 (Foundation for Statistical Computing, Vienna, Austria) and Stata Intercooled, Release 16. (StataCorp LLC College Station, TX).

## Results

### Demographics and disease characteristics

The mean age of the study participants was 30 years (range, 20–47 years) for younger adults and 57 years (range, 50–71 years) for older adults. Forty-eight percent of the participants were female and 48% were working. The majority (60%) of the participants had a severe SCD genotype (Hb SS or Sβ^0^). For disease-modifying therapies, 60% were on hydroxyurea and 18% were on chronic transfusion therapy. For pain management, 20% of younger adults and 35% of older adults were prescribed long-acting opiates, and 75% of younger adults and 85% of older adults were prescribed short-acting opiates. The most common SCD complications for all participants (lifetime prevalence) were acute chest syndrome/pneumonia (75%), avascular necrosis of any joint (60%), gallbladder disease requiring cholecystectomy (58%), and sickle cell retinopathy (40%). Demographics, disease characteristics, and therapies are summarized in Table 2.

**Table 2:** Patient demographic, disease characteristics, and therapies

Demographics	Younger (*n* = 20)	Older (*n* = 20)	All (*n* = 40)
Mean age in years (range)	30 (20–47)	57 (50–71)	44 (20–71)
Female, % (*n*)	45 (9)	50 (10)	48 (19)
Income ≤ $50,000, % (*n*)	45 (9)	50 (10)	48 (19)
Employment status, % (*n*)			
Working	45 (9)	50 (10)	48 19)
Unemployed	15 (3)	5 (1)	10 (4)
Disabled	20 (4)	30 (6)	25 (10)
Retired	0 (0)	10 (2)	5 (2)
Student	20 (4)	0 (0)	10 (4)
Other	0 (0)	5 (1)	3 (1)
Education level, % (*n*)			
High school graduate or less	20 (4)	20 (4)	20 (8)
Some college/associates/technical school	40 (8)	35 (7)	38 (15)
Bachelor’s degree	25 (5)	25 (5)	25 (10)
Advanced degree	15 (3)	20 (4)	18 (7)
Lives alone, % (*n*)	25 (5)	20 (4)	23 (9)
**Disease characteristics**			
SCD genotype, % (*n*)			
Hb SS	60 (12)	55 (11)	58 (23)
Hb Sβ^0^	5 (1)	0 (0)	3 (1)
Hb Sβ^+^	15 (3)	10 (2)	13 (5)
Hb SC	20 (4)	35 (7)	28 (11)
Hemoglobin (g/dL) mean ± SD, (Range)	9.8 ± 1.9 (6.6–13.9)	8.9 ± 2.4 (5.6–13.5)	9.3 ± 2.2 (5.6–13.9)
Strokes, % (*n*)	10 (2)	15 (3)	13 (5)
Sickle cell retinopathy, % (*n*)	25 (5)	55 (11)	40 (16)
History of acute chest syndrome/pneumonia, % (*n*)	85 (17)	65 (13)	75 (30)
Pulmonary hypertension, % (*n*)	0 (0)	25 (5)	13 (5)
Spleen removed, % (*n*)	35 (7)	25 (5)	30 (12)
Chronic kidney disease, % (*n*)	10 (2)	45 (9)	28 (11)
Cholecystectomy, % (*n*)	45 (9)	70 (14)	58 (23)
Iron overload, % (*n*)	25 (5)	20 (4)	23 (9)
Avascular necrosis of any joint, % (*n*)	40 (8)	80 (16)	60 (24)
Joint Surgery, % (*n*)	30 (6)	30 (6)	30 (12)
Leg ulcers, % (*n*)	5 (1)	15 (3)	10 (4)
Hypertension, % (*n*)	0 (0)	35 (7)	18 (7)
Diabetes, % (*n*)	0 (0)	5 (1)	3 (1)
Hearing (fair or poor), % (*n*)	0 (0)	25 (5)	13 (5)
**Healthcare Utilization**			
Hospitalized for pain in last year, % (*n*)	60 (12)	45 (9)	53 (21)
Visited the ED in the last year, % (*n*)	70 (14)	65 (13)	68 (27)
Visited Sickle Cell Day Hospital in last year, % (*n*)	35 (7)	47 (9)	41 (16)
≥ 4 hospitalizations in the last year, % (*n*)	0 (0)	10 (2)	5 (2)
*Severe pain crisis at home without hospitalization in last 6 months, % (*n*)	70 (14)	75 (15)	73 (29)
**Sickle cell disease medications and therapies**			
Hydroxyurea use, % (*n*)	65 (13)	55 (11)	60 (24)
Chronic transfusion therapy, % (*n*)	20 (4)	15 (3)	18 (7)
Iron Chelation Therapy, % (*n*)	25 (5)	15 (3)	20 (8)
Long-acting opiates, % (*n*)	20 (4)	35 (7)	28 (11)
Short-acting opiates, % (*n*)	75 (15)	85 (17)	80 (32)

### Feasibility results

**Fig. 1:**
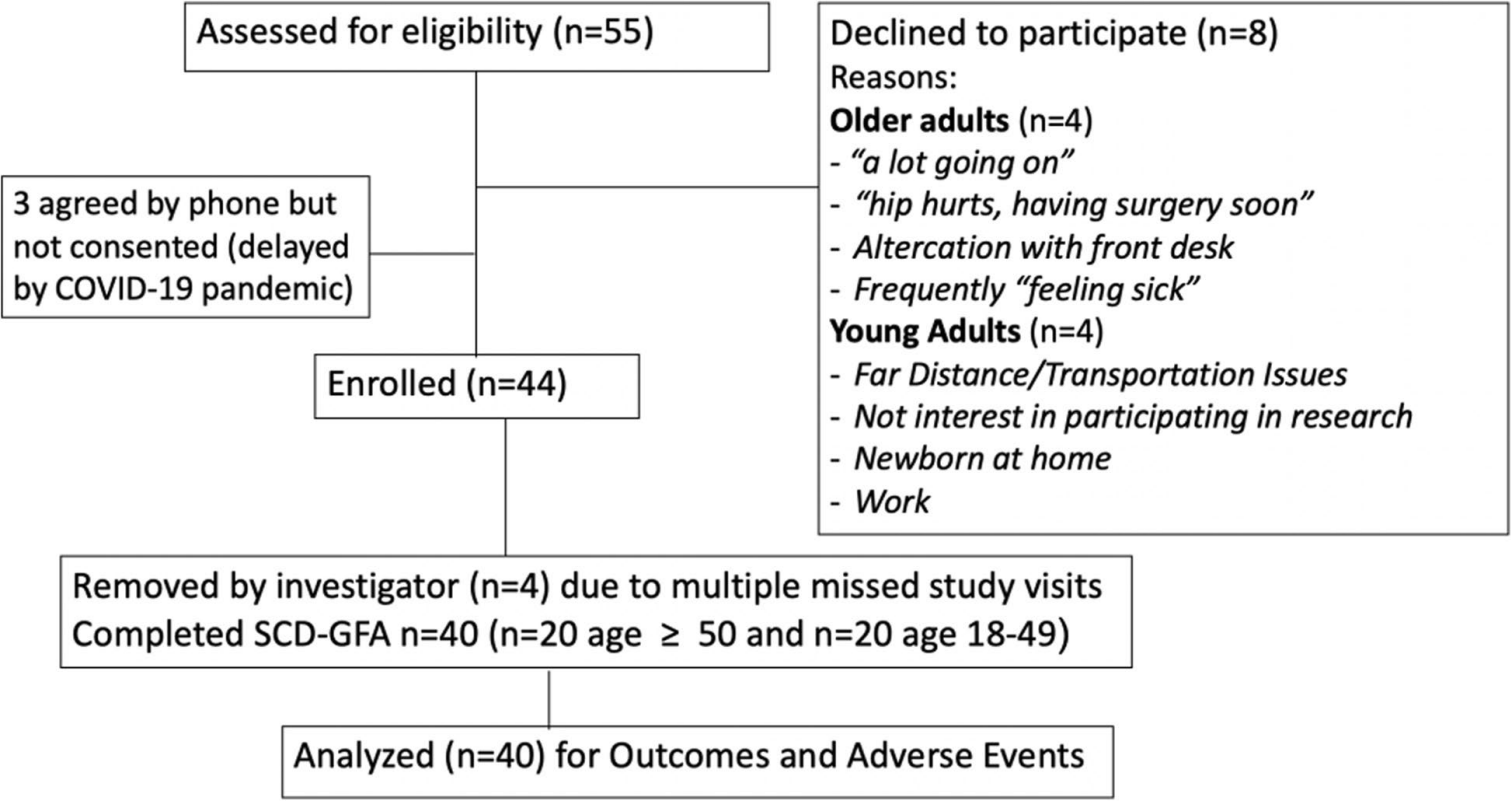
Consort diagram. We approached 55 participants for the study, consented 44, removed 4 due to multiple missed study visits, and performed the Sickle Cell Disease Functional Assessment in 20 younger adults and 20 older adults. Reasons participants gave for declining to participate are listed

Eighty percent of patients approached for the study consented, and 91% of consented participants completed the SCD-FA. The median duration of the assessment was 89 min (IQR 80–98 min), and there were no adverse events during or after the SCD-FA was performed (Fig. 1).

On the acceptability survey, 95% reported that the length of the SCD-FA was appropriate. One participant found a question upsetting, which was about a history of drug use. Five percent reported that there was at least one item on the questionnaires that was difficult to understand. When asked about feedback on removing items from the SCD-FA, 10% recommended removing the MoCA due to difficulties performing certain portions.

### Functional status

Thirteen percent of all participants were dependent in 1 or more ADL/IADL (20% of younger adults vs. 5% of older adults). Only 5% of all participants had a patient-reported KPS < 80% and 2.5% had a physician-reported KPS < 80% (KPS ≥ 80% indicates good performance status [30]). There was more variability on the Physical Functioning subscale of the 36-item Short Form Health Survey (SF-36) with a mean score for all participants of 77 (SD = 20) and a range of 25–100 on a scale of 0–100 [31]. Thirteen percent (5) of participants had a fall in the last 6 months (≥ 1 fall indicates an increased risk of subsequent falls [16]).

**Fig. 2:**
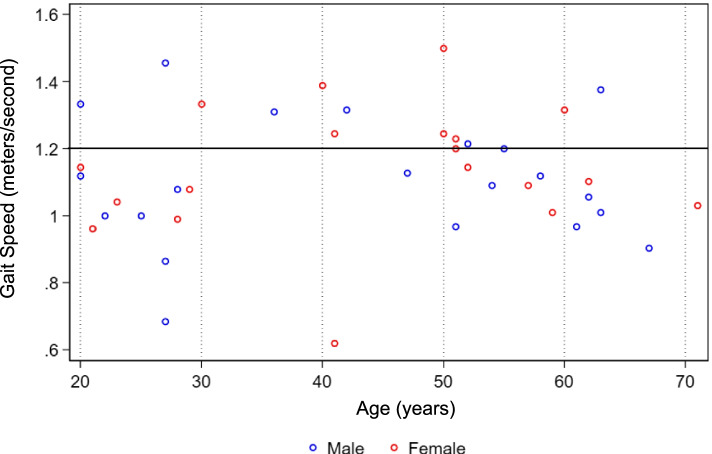
Usual gait speed (meters/second) by age and gender among individuals with sickle cell disease The figure displays the fastest of 2 usual gait speed trials stratified by age decade. The mean gait speed for all participants was 1.12 m/s (95% CI 1.06, 1.18). The majority of participants (63%) had a gait speed slower than 1.2 m/s (the speed necessary to safely cross the street at an intersection). Twenty-five percent had a gait speed slower than 1 m/s

The results of the physical performance measures with the expected performance based on normative values for their age and gender and their equivalent functional age is summarized in Table 3. For usual gait speed, younger adults had a mean walking speed of 1.10 (SD = 0.22) and older adults had a mean walking speed of 1.14 m/s (SD = 0.15). Usual gait speed by age shown in Fig. 2 demonstrates that the majority (63%) of both younger and older males and females had a slow usual gait speed (< 1.2 m/s) [32]. Gait speed did not significantly decrease with age. On the TUG, younger adults had a mean time of 9.2 seconds (SD = 2.2 s) and older adults had a mean time of 10.1 s (SD = 1.7). Sixty-eight percent of participants had a slow TUG time > 10 s and 10% of participants had a TUG > 12 s [33]. Both younger and older adults had a mean TUG time that was longer compared to the general population when stratified for age and gender (Table 3) [22, 27].

**Table 3:** Results of physical performance measures for younger adults (age 18–49, mean age 30; *N* = 20) and older adults (age ≥ 50, mean age 57; *N* = 20)

		Actual scores Mean ± SD	Performance for healthy norms by age and gender	Equivalent functional age (years)
**Gait speed** [26] **(m/s)**	**Age 18–49**	Male: 1.12 ± 0.23	Males: 1.46 ± 0.9	Males: 80–89
		Female: 1.09 ± 0.23	Female: 1.42 ± 1.3	Females: 80–89
	**Age ≥ 50**	Male: 1.09 ± 0.14	Males: 1.36 ± 2.1	Males: 80–89
		Female: 1.19 ± 0.15	Female: 1.30 ± 2.1	Females: 80–89
**Timed Up Go** [22, 27] **(s)**	**Age 18–49**	Male: 8.9 ± 1.6	Males: 8.6 ± 1.2	Males: 70–79
		Female: 9.5 ± 2.8	Females: 8.6 ± 1.2	Females: 70–79
	**Age ≥ 50**	Male: 10.7 ± 2.0	Males: 8.0 ± 2	Males: 80–89
		Female: 9.5 ± 1.1	Females: 8.0 ± 2	Females: 80–89
**30-second chair stand** [28] **(#)**	**Age 18–49**	Male: 15 ± 5	Males: N/A	Males: 65–69
		Female: 13 ± 5	Females: N/A	Females: 65–69
	**Age ≥ 50**	Male: 10 ± 4	Males: 16 ± 4.3	Males: 85–89
		Female: 12 ± 3	Females: 15 ± 4.0	Females: 75–79
**6-min walk** [22, 29] **(m)**	**Age 18–49**	Male: 585 ± 104	Male: 638 ± 44	Males: 60–69
		Female: 498 ± 118	Female: 593 ± 57	Female: 60–69
	**Age ≥ 50**	Male: 465 ± 73	Male: 572 ± 92	Males: 80–84
		Female: 499 ± 64	Female: 538 ± 92	Female: 70–74

**Fig. 3:**
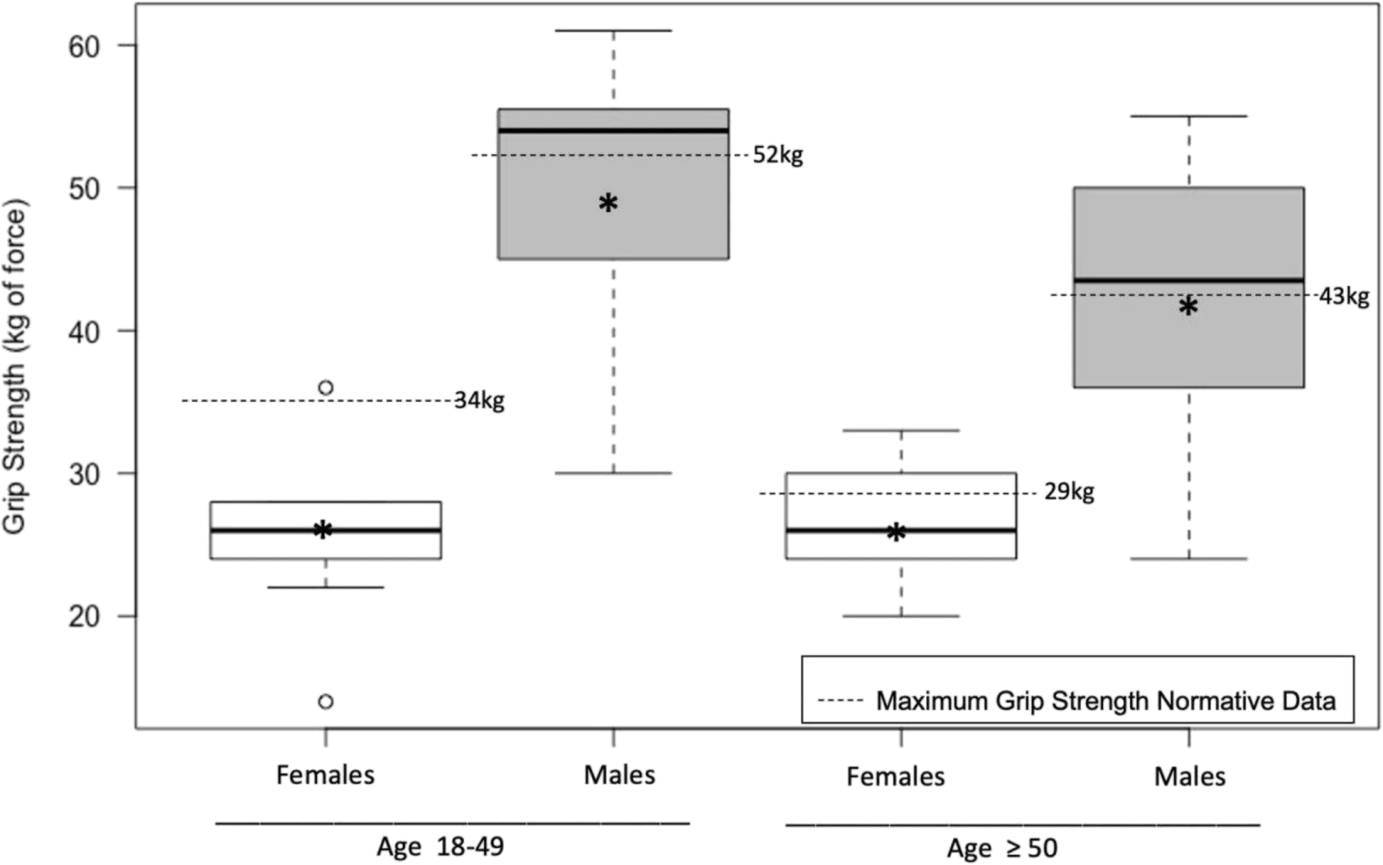
Maximum seated grip strength by age group and gender in individuals with sickle cell disease. Boxplot of maximum seated grip strength of 3 trials on each hand. Mean grip strength by age group and gender is also represented by the star on each boxplot and compared to mean maximum grip strength normative values by age and gender in the United States published by Hanten et al. [35]

On the 6MWT younger adults walked a mean distance of 546 meters (SD = 116) and older adults walked a mean distance of 482 meters (SD = 69) (Based on American Society of Hematology guidelines, a 6MWT distance < 333 m requires further evaluation for pulmonary hypertension in SCD [34]). On the 30-second chair stand test, younger adults completed a mean of 14 chair stands (SD = 5) and older adults completed a mean of 11 chair stands (SD = 4). For the 6MWT and the 30-second chair stand, younger adults had performance similar to people in their 60s and 70s in the general population and older adults had performance similar to people in their 70s and 80s (Table 3) [22, 28, 29]. There were expected gender difference in seated grip strength with mean maximum grip strength of 26 kg of force (SD 5.8) for younger females, 49 kg (SD 10.7) for younger males, 26 kg (SD 4.3) for older females, and 42 kg (SD 10.9) for older males (Fig. 3) [35–37].

### Cognitive functioning

On cognitive testing, the mean MoCA score for all participants was 26 (SD = 3). Thirty-five percent of younger adults and 50% of older adults had a MoCA score consistent with cognitive impairment (score < 26 out of 30). When using score < 23, which is a suggested optimal cutoff for non-Hispanic African Americans, 15% of younger adults and 25% of older adults had scores < 23 [38]. Out of all participants, only 5% (2) had a score greater than 9 (concerning for cognitive impairment) on the Blessed Orientation Memory Concentration Test.

### Weight status

The mean body mass index (BMI) for all participants was 26 kg/m^2^ (SD = 5). Eighteen percent had a BMI categorized as obese (≥ 30 kg/m2) and 18% had a low BMI (< 22 kg/m2). Thirteen percent reported having unintentional weight loss in the last 6 months.

### Psychosocial measures

There was a wide range of results for the Medical Outcome Study Social Support Survey instrument with a mean score for all participants of 70 (SD = 21) and a range of 7–93 on a scale of 0–100 (with lower scores indicating less support). Older adults had a mean social support score of 72 (SD = 25) and younger adults had a mean score of 69 (SD = 18). Eighteen percent of all participants reported that their health interfered with their social activities at least some of the time in the last month, 35% reported that their social activity decreased in the last 6 months due to their condition, and 38% reported that their social activity was limited compared to their others their age because of their health. On the 18-item Mental Health Inventory (MHI-18), the mean score for all participants was 78 (SD = 14) with a range of 27–100 on a scale of 0–100 (with higher scores indicating better mental health). Younger adults had a mean mental health score of 77 (SD = 16) with a range 27–100, and older adults had a mean score of 79 (SD = 12) with a range 43–97. On the anxiety subscale of the MHI-18, 60% of younger adults and 20% of older adults had a score ≤ 75. On the depression subscale of the MHI-18, 30% of younger adults and 20% of older adults had a score ≤ 75.

### Floor and ceiling effects and burden

We assessed each measure for floor and ceiling effects and assessed burden based on results of the patient acceptability survey and length of each measure. In Table 4, we described the mean scores for each patient-reported measure, range of participant scores, the upper and lower limit scores of each measure for reference, and percent of participants with floor or ceiling effects. If > 15% of participants had floor or ceiling effects, we considered this as clinically significant. We described the action taken to either keep or remove the measure based on floor and ceiling effects and/or respondent and administrative burden.

**Table 4:** Floor and ceiling effects of patient-reported outcome measures in the Sickle Cell Disease Functional Assessment (*N* = 40)

Measure	Mean ± SD	Range of participant scores	Lower and upper limits of measure	% (n) with floor effect*	% (n) with ceiling effect*	Action taken
OARS ADL/IADL	28 ± 1.4	20–28	0–28	0 (0%)	88% (35)*	Will keep measure since it has low respondent burden and is an important marker of functional dependence in older adults in the general population. Will reassess floor and ceiling effects and validity in a larger sample of adults with SCD
Physical Functioning Subscale of SF-36	77 ± 20	25–100	0–100	0% (0)	10% (4)	Keep measure
KPS – patient reported	89 ± 11	60–100	0–100	0% (0)	38% (15)*	Remove measure due to ceiling effect and redundancy with other measures in the functional status domain
KPS – physician reported	92 ± 9	60–100	0–100	0% (0)	50% (20)*	Remove measure due to ceiling effect and redundancy with other measures in the functional status domain
Falls/6 months	0.2 ± 0.6	1–3	> 0	88% (35)*	N/A	Keep measure despite floor effect given low respondent burden. Will need to assess the incidence of falls and validity of the measure in a larger sample of adults with SCD
MHI-18	78 ± 14	27–100	0–100	0% (0)	3% (1)	Remove due to issues with length and interpretability. Replace with PHQ-9 and GAD-7 for shorter length, familiarity with use in adults with SCD, and better interpretability
MOS Social Support Survey	70 ± 21	7–93	0–100	0% (0)	0% (0)	Keep measure
BOMC Test	3.2 ± 3.6	0–14	0–28	40% (16)*	0% (0)	Remove measure due to floor effect
MoCA	26 ± 3	19–30	0–30	0% (0)	7.5% (3)	Remove measure due to respondent and administrative burden. Replaced with Mini-Cog

*ADL/IADL* Older Americans Resources and Services Activities of Daily Living/Instrumental Activities of Daily Living, *SCD* sickle cell disease, *SF*-*36* 36-item Short Form, *KPS* Karnofsky Performance Status, *MHI*-*18* 18 Item Mental Health Inventory, *MOS* medical outcome study, *BOMC* Blessed Orientation-Memory-Concentration Test, *MoCA* Montreal Cognitive Assessment

*Floor and ceiling effects of more than 15% are considered to be significant

## Discussion

The results of this study show that the SCD-FA is feasible, acceptable, and safe in adults with SCD. The participants found the length to be acceptable and even had suggestions for additional measures to add.

SCD-FA is a multidimensional assessment tool that includes validated measures adapted from a cancer-specific geriatric assessment with additional measures that focus on health issues at the intersection of SCD and geriatrics. Similar to the oncology geriatric assessment developed by Hurria et al., a large portion of our participants had no deficits in their ADL/IADL at baseline [16, 39]. The results of the SF-36 Physical Functioning subscale were highly variable compared to ADL/IADL scores since it measures a wider variety of physical demands that individuals of all ages may experience throughout the day, such as moving heavy objects and climbing stairs. In addition, our participants had similar baseline scores on Physical Functioning subscale to individuals without SCD in the African American Health Study (77, SD = 20 in our population vs 80.81, SD 25 for non-SCD) [40]. Both ADL/IADL and the SF-36 Physical Functioning subscale may have a valuable role in defining dependence and functional limitations in patients with SCD since they are a heterogenous population with varying levels of capabilities depending on their disease state.

The SCD-FA differs from geriatric assessments used by many other specialties and differs from assessment tools currently used by SCD providers in that it includes multiple physical performance measures. Both objective and subjective measures of physical function play vital roles in assessing different aspects of physical health and both have predictive validity in the general population [10, 41]. The strength of including subjective measures is that they provide important information about what a person experiences and their perception of their health. Objective measures provide observable data on a person’s capabilities and functional impairments. SCD providers and patients often have concerns about function and disability since SCD causes significant musculoskeletal complications and pain that can limit mobility. These issues often go unrecognized until impairment is advanced, which makes the SCD-FA a desirable assessment tool since it includes both patient-reported and objective measures of function.

When we compared our physical performance results to the normative values in the general population, we found that our participants had a physical performance similar to individuals 20–30 years older than their chronological age [22, 26–29]. Our participants may have had poorer performance compared to age and gender matched normative data since most individuals with SCD have anemia and experience vaso-occlusive events that affect multiple organ systems. These complications include avascular necrosis of the joints, pain episodes, cardiopulmonary disease, and neurologic complication such as silent and overt strokes [4, 42]. A study in children and adolescents with SCD showed that individuals with SCD have lower levels of physical activity compared to healthy controls measured by accelerometer [43]. In a study of maximal exercise testing in adults with SCD, older age, female sex, lower hemoglobin, higher BMI, and lower percent predicted forced expiratory volume in 1 second were independently associated with lower exercise capacity [44]. Another study on 6MWT in children with SCD showed that low hemoglobin, low fetal hemoglobin, and low red cell deformability were independent predictors of low 6MWT performance [45]. The majority of our participants had a usual gait speed slower than the speed required to safely cross the street at an intersection (1.2 m/s), and 25% had a gait speed slower than 1 m/s, which has been associated with increased mortality in the general population [32, 46]. The majority of participants had a TUG > 10 s, which has been associated with fractures in older adults [47, 48]. Ten percent of our participants had a TUG > 12 s, which has been associated with an increased risk of falls in community-dwelling older adults [49]. Prior studies on the 6MWT and SCD, which mainly include younger populations, have participants that also demonstrate functional impairment; however, these studies are limited in that they did not measure balance, gait speed, and strength [50, 51]. Including additional measures, such as gait speed, TUG, 30-second chair stand, and grip strength may provide detailed information about a patient’s capabilities, such as deficits in upper body strength (grip strength), deficits in lower body strength (30-second chair stand), balance deficits (TUG), and deficits in aerobic endurance (6MWT). These measures may better guide interventions to address specific deficits [52, 53].

Although this study was not powered to detect differences in function based on age or correlations between individual measures, there were notable trends. We found that older adults had a shorter 6MWT distance, more cognitive impairment, fewer chair stands, and more chronic complications such as avascular necrosis and renal disease compared to younger adults. Younger adults trended toward having more hospitalizations and higher anxiety and depression scores on the MHI-18. In our study population, time to complete the TUG was not remarkably different between younger and older adults, and usual gait speed did not significantly decrease with age as occurs in the general population. This similarity in performance could possibly be explained by the small sample size and the fact that in our study, most participants in the younger and older adult groups had anemia, pain episodes, and musculoskeletal complications. Both groups reported having severe vaso-occlusive pain crises at home in the last 6 months (≥ 70% in both groups), several participants in both groups had pain crises requiring emergency room visits and/or hospitalizations in the last year (≥ 45%), and the majority of participants in both groups reported taking short-acting opioids (≥ 75% in both groups). There were also high rates of avascular necrosis of any joint in both groups (40% of young adults and 80% in older adults) with 30% of younger adults and 30% of older adults requiring joint surgery. Recurrent acute pain episodes, anemia, and early onset avascular necrosis may impact mobility, which could explain why younger adults have similar performance on gait speed and TUG test as older adults [54]. In addition, there are differences in survival with only the healthiest adults living into their 50s and 60s. Further comparative studies with larger sample sizes are required to determine factors that impact physical performance in each age group. These measures will also need to be evaluated in a larger study to determine clinically significant cut points for adults with SCD.

### Limitations

There are limitations to this study. First, there may be a selection bias given that participants are individuals who come to a SCD clinic in an academic center, were willing to participate in research studies, and able to meet the inclusion and steady-state criteria. Secondly, pain and SCD complications that are active at the time of the assessment may interfere with performance. These SCD complications can confound the results of measures, such as physical performance tests and psychosocial measures. Many SCD complications increase with age; therefore, with older participants, it becomes increasingly important to determine how pain and other specific complications affects results [4]. Lastly, there are limitations to interpretation of the results based on normative values available in the literature since many normative studies include few or no African Americans. Also, SCD-specific cut points have not been established. To address this, we have made every effort to compare results to populations that include a more diverse population. During validation of the SCD-FA, we will set cutpoints for individuals with SCD at levels that are predictive of relevant outcomes.

### Optimization

Our assessment took three times longer to complete compared to the oncology geriatric assessment due to additional performance-based physical and cognitive measures and SCD-specific questions [16]. Based on the results of this pilot study, we revised the SCD-FA to develop a briefer assessment to enhance its usability. We removed the Blessed Orientation Memory Concentration Test and the KPS due to floor and ceiling effects. We replaced the MoCA with the Mini-Cog© due to high respondent burden based on the length of the MoCA and difficulties understanding the questions. We did not remove the ADL/IADL and the question about number of falls despite them having floor and ceiling effects in order to test them further in a larger study. ADL/IADL and number of falls are both measures with low respondent burden and in multiple studies have been shown to be predictive of mortality in older adults in the general population [55–58]. In the next phases of this study, we will begin to validate the revised version of the SCD-FA, continue to refine measures based on patient and provider interviews, and determine its ability to predict important outcomes, such as functional dependence and mortality. Given the paucity of validated functional assessment tools available to assess older adults with SCD, we expect that the SCD-FA will be a valuable tool used to improve management of this vulnerable population by (1) characterizing the capabilities and physiologic age of individuals with SCD; (2) risk stratifying patients by determining which individuals are at highest risk for functional decline, dependence, and mortality; (3) identifying targets for interventions that have been successful in geriatrics [52]; and (4) and assessing response to curative therapies and novel SCD-specific therapeutics.

## Conclusion

In summary, the SCD-FA is feasible, acceptable, and safe in both older and younger adults with SCD. This pilot study provides important information to refine this tool to best suit the needs of adults with SCD. The SCD-FA will undergo further refinement and validation in a larger study. In this small sample, the SCD-FA was able to detect functional impairments in multiple measures, such as slow gait speed and TUG, which are measures associated with important health outcomes in general population and in multiple other chronic disease states, such as patients with hematologic malignancies and advanced renal disease. The physical measures of the SCD-FA also permit the comparison of their physical performance to specific age groups in the general population. Functional assessments serve an important role in the care of individuals with SCD by providing a framework for identifying and intervening on impairments in multiple dimensions of health. This supports our goal of improving the quality in addition to the quantity of life for people with SCD.

## Data Availability

The datasets used and/or analyzed during the current study are available from the corresponding author on reasonable request.

## Abbreviations

SCD-GFA: Sickle Cell Disease Geriatric Functional Assessment
SCD: Sickle cell disease
6MWT: Six-minute walk test
ADL/IADL: Activities of Daily Living and Instrumental Activities of Daily Living
CGA: Comprehensive geriatric assessment
HPLC: High-performance liquid chromatography
ED: Emergency department
PhenX: Phenotypes and eXposures
CONSORT: Consolidated Standards of Reporting Trials
TUG: Timed-Up-And-Go
KPS: Karnofsky Performance Status
MoCA: Montreal Cognitive Assessment
SF-36: 36-item Short Form Health Survey
BMI: Body mass index
MHI-18: 18-item Mental Health Inventory
